# Evaluation of human exposure to parabens in north eastern Poland through hair sample analysis

**DOI:** 10.1038/s41598-021-03152-8

**Published:** 2021-12-08

**Authors:** Joanna Wojtkiewicz, Manolis Tzatzarakis, Elena Vakonaki, Krystyna Makowska, Slawomir Gonkowski

**Affiliations:** 1 Department of Pathophysiology, School of Medicine, Collegium Medicum, University of Warmia and Mazury, 10-900 Olsztyn, Poland; 2 Laboratory of Toxicology Science and Research, Medicine School, University of Crete, 70013 Heraklion, Crete Greece; 3 Department of Clinical Diagnostics, Faculty of Veterinary Medicine, University of Warmia and Mazury in Olsztyn, Oczapowskiego 14, 10-957, Olsztyn, Poland; 4 Department of Clinical Physiology, Faculty of Veterinary Medicine, University of Warmia and Mazury in Olsztyn, Oczapowskiego 13, 10-957, Olsztyn, Poland

**Keywords:** Biomarkers, Medical research, Risk factors

## Abstract

Parabens (PBs) are a group of substances commonly used in industry. They also pollute the environment, penetrate into living organisms and adversely affect various internal organs. During this study, the degree of exposure of people living in Olsztyn, a city in north eastern Poland, to selected parabens most often used in industry was studied. The chemicals under investigation included: methyl paraben—MePB, ethyl paraben—EtPB, propyl paraben—PrPB, benzyl paraben BePB and butyl paraben -BuPB. To this aim, hair samples collected from the scalps of 30 volunteers were analyzed using a liquid chromatography–mass spectrometry technique. All PBs studied were present in a high percentage of analyzed samples (from 76.7% in the case of BePB to 100% in the case of MePB and PrPB). The mean concentration levels were 4425.3 pg/mg for MeBP, 704.0 pg/mg for EtPB, 825.7 pg/mg for PrPB, 135.2 pg/mg for BePB and 154.5 pg/mg for BuPB. Significant differences in PB concentration levels between particular persons were visible. On the other hand, gender, age and artificial hair coloring did not cause statistically significant differences in PB levels. Obtained results have clearly indicated that people living in north eastern Poland are exposed to various PBs, and therefore these substances may affect their health status. However, the evaluation of PBs influence on human health requires further research.

## Introduction

Parabens (PBs) are a large group of organic substances, which are the alkyl esters of parahydroxybenzoic acid, that differ among themselves by their functional groups^1^. PBs may be synthesized by some species of microbes and plants, but more often there are synthetic parabens commonly used in various branches of industry since the 1930s^1,2^. These substances play an important role in food, chemical, and cosmetics production, and they are included into many everyday objects, such as personal care products, shampoo, lipsticks, food packaging, baby wipes, drugs and many others^1,3,4^. PBs with short-chain functional groups, such as methyl paraben (MePB), ethyl paraben (EtPB), propyl paraben (PrPB) and butyl paraben (BuPB) are especially often used in the industry^1^.

PBs penetrate from the above-mentioned objects into the environment. Up to now, these substances have been detected in different parts of the world in water (both tap and surface), human food, animal food, soil and dust^5–10^). PBs can also enter living organisms through the respiratory tract, gastrointestinal systems, skin and placenta (in utero)^11,12^. Previous studies have reported the presence of PBs in human blood, urine, seminal plasma, tissues and hair^13,14^.

For decades PBs were considered to be completely safe for living organisms, but since the end of the twentieth century more and more studies have reported their adverse effects on humans and animals. Currently PBs are classified as endocrine disruptors, which have genotoxic and cytotoxic effects^15,16^ and affect many internal organs and systems, mainly including the reproductive, immune and endocrine systems^17–20^. Some studies have also reported correlations between PBs and obesity, disorders in the nervous system developmental disorders and neoplastic processes^11,21–23^. Due to adverse effects of PBs, the legislation of many countries has introduced the limitation of these substances. The permitted content of PBs in cosmetics in the European Union amounted to 0.4% for one PB and 0.8% for all PBs^24^.

Due to the negative multidirectional impact of PBs on human health described in recent years, as well as the widespread presence of these compounds in the human environment, the monitoring of human exposure to parabens by determining their levels in the tissues and fluids of the body has become an important problem in modern toxicology. Until now, the human exposure to PBs has been studied in various parts of the world, and the concentration levels of these compounds clearly depends on the part of the world, industrialization and environmental pollution in the place where the observations were conducted. Furthermore, exposure depends on the degree of consumption of cosmetic products by the studied population, as well as the type of paraben and the matrix tested^13,14^. Previous studies have shown that PBs with a short-chain functional group, such as MePB, EtPB and/or PrPB, are present in the human body in higher concentrations than other PBs^13^. The concentration of MePB (the PB observed in the human body in the highest concentration) fluctuates from 0.8 ng/g in human breast milk in the USA^25^ to 14,187 ng/g in the hair in Spain^13^, according to the literature. However the knowledge of human exposure to PBs in Poland is extremely scanty and limited to a description of PBs present in human urine^26–28^.

It should be pointed out that an analysis of the hair for the presence of PBs is a relatively new method in toxicology. Previous observations have shown that hair is a suitable matrix to study long term exposure of living organisms to a broad spectrum of toxic substances occurring in the environment^13,29,30^. Moreover, it is known that hair analysis can replace studies on “classic” matrices, such as blood or urine, because it has similar reliability and sensitivity^31^. Simultaneously, the collection of hair samples is easy and completely stress-free, and such samples may be easily stocked and shipped over long distances. It is also known that hair analysis is suitable for the evaluation of exposure to PBs^13,14^.

Therefore, this study is the first description of an analysis of the hair of Polish residents to simultaneously assess exposure to the most commonly used industrial parabens, such as MePB, EtPB, PrBP, BuPB and BePB using hair samples analysis.

## Results

**Figure 1:**
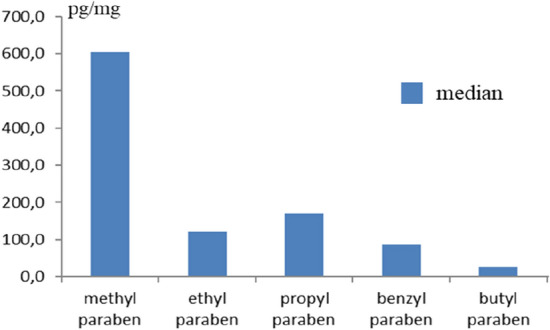
Comparison of median values of parabens included in the present study.

During the present study, PBs were detected in all hair samples. MePB and PrPB have been noted in all samples studied. In the case of EtPB, the positive percentage was 96.7%. Slightly lower detection rates were noted for BuPB and BePB, where these values amounted to 90% and 76.7%, respectively. The highest concentration levels were noted in the case of MeBP. The mean concentration level of this substance achieved 4425.3 ± 10,281.8 pg/mg. The mean concentrations of other PBs studies were significantly lower and did not exceed 1000 pg/mg. These values amounted to 825.7 ± 2166.0 pg/mg, 704.0 ± 1775.4 pg/mg and 154.5 ± 501.1 pg/mg for PrPB, EtPB and BuPB, respectively. During the present study the lowest concentration level was observed in the case of BePB, which had a mean concentration of 135.2 ± 144.5 pg/mg. Extreme differences in concentration levels of PBs have been noted between particular volunteers included in the study. These differences were visible in the case all PBs studied, but the largest range of concentration levels concerned MePB (from 87.2 to 42,430.1 pg/mg). Concentration levels of PBs observed during the present study are summarized in Table 1 and illustrated in Fig. 1 (median values).

**Table 1:** Concentration levels of parabens (pg/mg) noted in the hair samples from 30 volunteers (one sample from each volunteer) during the present study.

Sample number	Methyl paraben	Ethyl paraben	Propyl paraben	Benzyl paraben	Butyl paraben
P1	669.2	282.3	448.6	39.4	49.3
P2	1794.4	288.3	489.7	157.5	211.7
P3	701.7	93.6	483.3	123.1	12.6
P4	791.4	447.3	58.9	36.0	99.3
P5	87.2	19.1	24.5	ND	4.6
P6	556.2	59.7	32.7	40.2	10.3
P7	663.3	52.5	22.0	ND	8.1
P8	42,430.1	4238.2	653.6	49.3	3.8
P9	682.8	175.7	25.7	ND	22.1
P10	597.9	369.0	717.6	93.0	19.8
P11	384.2	67.1	53.9	87.2	38.2
P12	10,661.7	340.2	80.0	75.1	185.5
P13	207.7	58.2	89.8	ND	59.0
P14	34,862.4	308.3	11,150.3	233.8	22.4
P15	371.7	38.4	160.9	ND	12.9
P16	610.1	608.8	21.4	67.7	4.1
P17	23,704.7	8413.1	3582.8	121.2	2636.0
P18	95.4	ND	18.9	188.5	ND
P19	182.8	26.1	43.2	ND	ND
P20	3535.6	202.1	782.5	356.9	281.0
P21	118.7	246.8	4077.8	700.7	45.3
P22	645.3	26.4	264.4	52.2	17.0
P23	492.0	104.7	165.7	117.9	ND
P24	204.1	120.7	207.9	74.3	132.8
P25	151.3	25.8	48.7	193.2	23.1
P26	679.3	36.1	173.2	63.1	32.3
P27	551.7	47.1	205.6	103.2	13.7
P28	182.3	47.2	62.1	ND	27.8
P29	5669.7	123.3	228.5	66.9	151.4
P30	472.9	3550.0	396.9	69.8	46.3
**Cumulative data**					
Median	604.0	120.7	169.5	87.2	27.8
Mean	4302.0	704.0	825.7	135.2	154.5
± SD	10,281.8	1775.4	2166.0	144.5	501.1
MIN	87.2	19.1	18.9	36.0	3.8
MAX	42,430.1	8413.1	11,150.3	700.7	2636.0
LOD (pg/mg)	0.4	1.0	0.7	1.1	0.2
LOQ (pg/mg)	1.4	3.3	2.2	3.6	0.8
% positive	100.0	96.7	100.0	76.7	90.0

LOD, limit of determination; LOQ, limit of quantification; ND, not detected.

**Figure 2:**
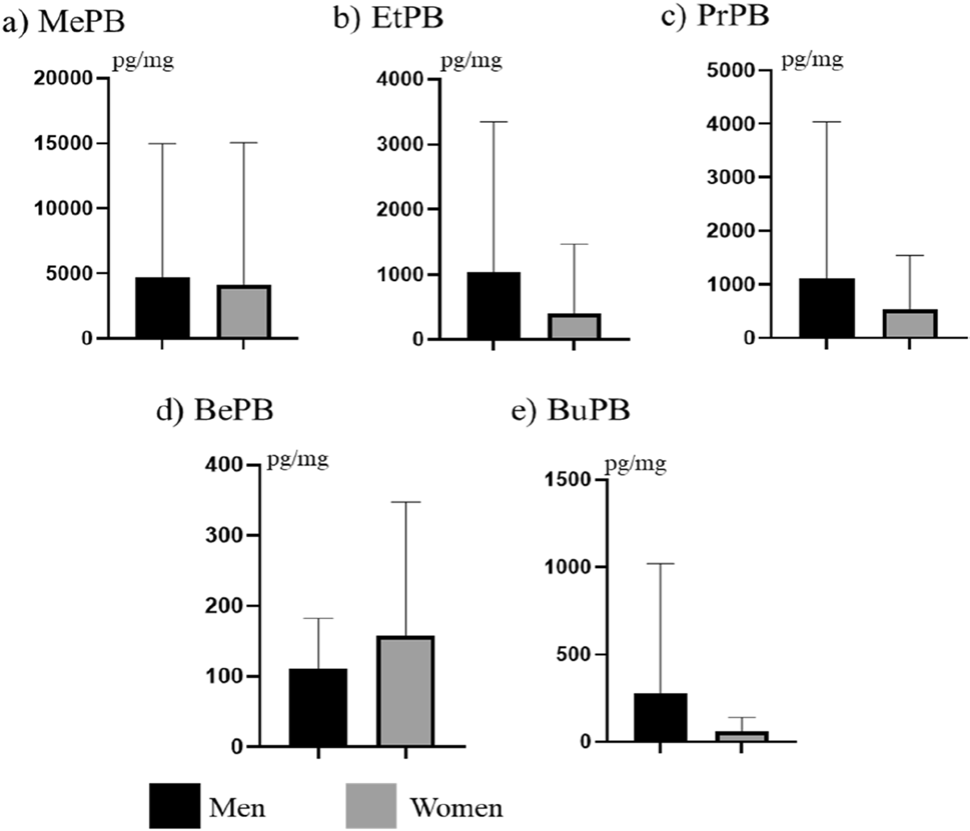
Mean concentration levels (± SD) of parabens in hair of men and women.

During the present study some differences in the concentration levels of PBs studied were observed between males and females (Fig. 2). In males, mean concentration levels (± SD) amounted to 4731 ± 10,289 pg/mg for MePB, 1035 ± 2309 pg/mg for EtPB, 1115 ± 2918 pg/mg for PrPB, 110.4 ± 71.68 pg/mg for BePB and 274.1 ± 746.6 pg/mg for Bu PB. In women, these values achieved 4137 ± 10,931 pg/mg, 403.3 ± 1067 pg/mg, 536 ± 1012 pg/mg, 158 ± 189.5 pg/mg, and 58.73 ± 79.82 pg/mg, respectively Differences in concentration levels of all PBs studied between men and women were not statistically significant.

**Figure 3:**
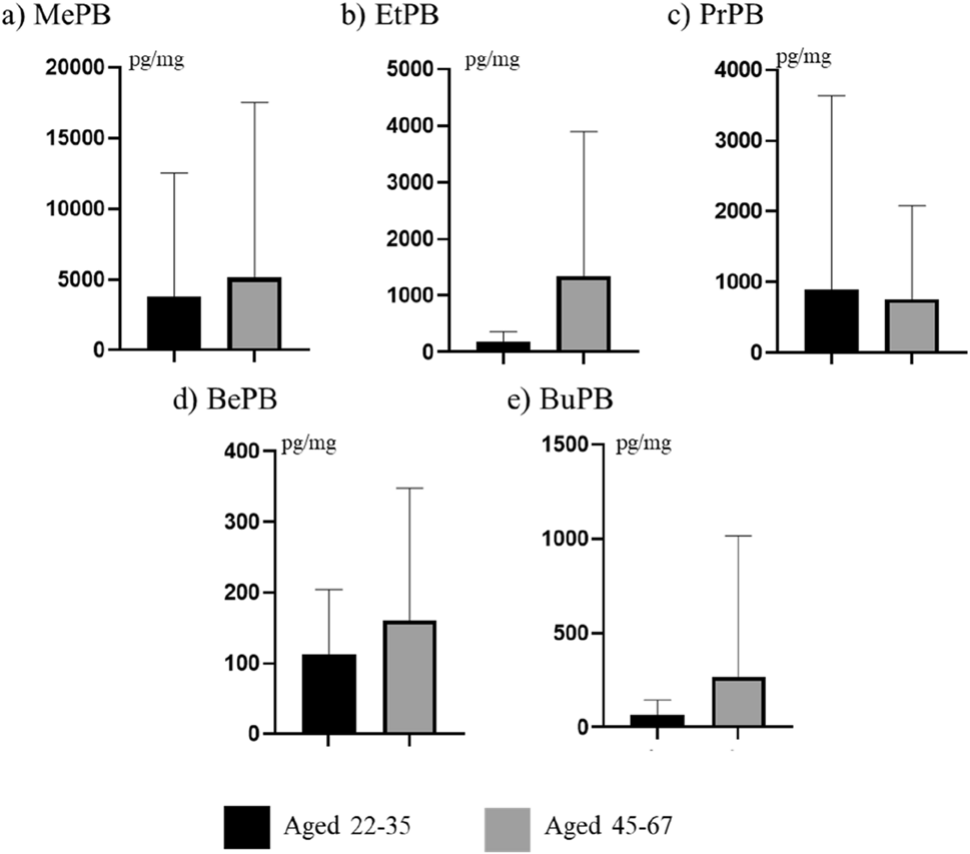
Mean concentration levels (± SD) of parabens in persons at the age of 22–35 and 45–67.

During the present study volunteers were also divided into two groups depending on age. The boundary between the groups was set at 35 as this age has traditionally been considered the end of adolescence. At this age, people often change their lifestyle and the aging processes in the body increase. Some differences in concentration levels of PBs were visible between volunteers at the age of 22–35, and persons at the age of 45–67 (Fig. 3). In the first group, the mean concentrations amounted to 3798 ± 8743 pg/mg for MePB pg/mg, 186.4 ± 177.7 pg/mg for EtPB, 8857 ± 2748 pg/mg for PrPB, 112.2 ± 92.77 pg/mg for BePB and 63.75 ± 81.52 pg/mg for BuPB.

In older volunteers, mean concentration levels of MePB, EtPB, BePB and BuPB were higher and achieved a level of 5143 ± 12,395 pg/mg, 1341 ± 2553 pg/mg, 160.4 ± 187.5 pg/mg and 267.8 ± 748.2 pg/mg, respectively. In turn, the concentration level of PrBP was lower (757.1 ± 1320 pg/mg). However, none of these differences were statistically significant.

**Figure 4:**
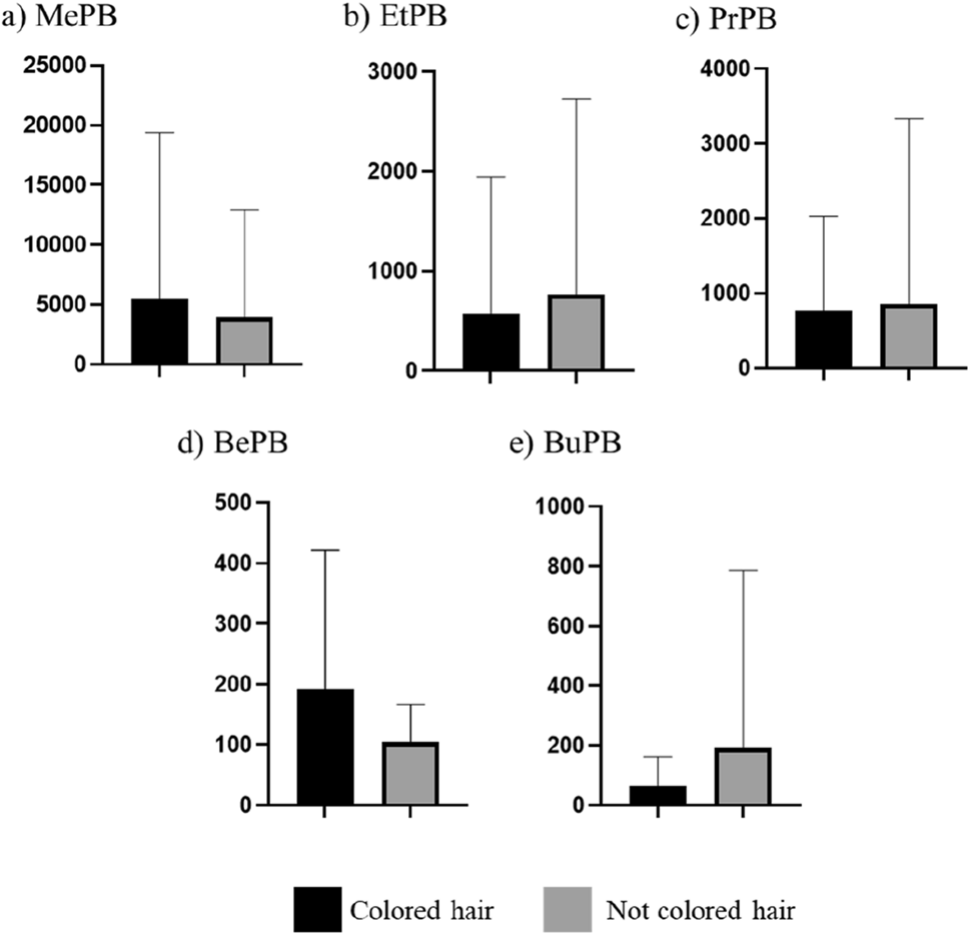
Mean concentration levels (± SD) of parabens in persons with colored hair and natural hair color.

During the present study, the comparison of the PB concentration levels in persons with colored hair and persons with natural hair color was also performed (Fig. 4). In persons with colored hair, the mean concentration levels amounted to 5497 ± 13,888 pg/mg for MePB, 569 ± 1378 pg/mg for EtPB, 758.9 ± 1269 pg/mg for PrPB, 192.2 ± 228.8 pg/mg for BePB and 66.61 ± 96.61 pg/mg for BuPB. In the group of persons with natural hair color, these values achieved 3966 ± 8943 pg/mg, 764.8 ± 1958 pg/mg, 854.3 ± 2481 pg/mg, 104 ± 61.56 pg/mg and 191 ± 595.2 pg/mg, respectively. These differences were also statistically insignificant.

## Discussion

Obtained results clearly show that people living in north eastern Poland are exposed to various PBs. The present study confirms previous observations that PBs are important environmental pollutants^4,32^. It should be underlined that in Poland, contrary to other countries, information about environmental pollution with parabens, as well as the degree of human exposure to these substances is relatively scanty and limited to only a few studies. It is known that PBs are present in the surface water in Poland^33–35^, and the largest concentration levels were observed in the case of MePB. The concentration levels of this substance in lake water may even achieve 1578 ng/L^33^. The concentration levels of other PBs in the surface water in Poland are considerably lower, because the maximum concentration levels noted in previous studies amounted to 349 ng/L, 165 ng/L and 23.6 ng/L for EtPB, PrPB and BuPB, respectively^33–35^. In turn, the relatively high concentration of PBs has been noted in wastewater in southern Poland, in which concentration levels of MePB, EtPB, PrBP and BuPB fluctuated within limits of 2235.0–40,898.6 ng/L, 791.2–8169.4 ng/L, 542.2–7803.3 ng/L and 68.8–710.7 ng/L, respectively^35^. It should be noted that pollution of the natural environment with PBs is correlated with the risk of human exposure to these substances. Therefore, the above-mentioned data confirm that the inhabitants of Poland are exposed to the adverse effects of parabens, not only contained, for example, in cosmetics, but also polluting the environment. Knowledge concerning biomonitoring of human exposure to PBs in people living in Poland are also extremely scanty and limited to only three studies^26–28^. These studies have described concentration levels of selected PBs in human urine. In women the mean concentration levels were 107.9 µg/L for MePB, 12.9 µg/L for EtPB, 18.67 µg/L for PrPB, 5.02 µg/L for BuPB and 2.8 µg/L for izobutylparaben (iPB)^28^. In men, concentration levels of the majority of PBs were slightly lower and depended on the study. According to one study, the average concentration of PBs in male urine amounted to 47.6 µg/L for MeBP, 8.6 µg/L for EtPB, 22.3 µg/L for PrBP, 1.4 µg/L for BuPB and 1.1 µg/L for iPB^26^, and according to a subsequent investigation, these values achieved 15.6 µg/L, 9.39 µg/L, 3.7 µg/L, 3.48 µg/L and 2.27 µg/L, respectively^27^.

However, it is relatively well established that the degree of human exposure to PBs clearly depends on the area where the studies have been performed^13,14,36,37^. For example, the levels of MeBP noted in human urine in Australia fluctuated from 74.4 to 1180 ng/g^36^, while in Brazil it was in the range of 0.82–26.15 ng/g^37^. Extreme differences in the concentration of PBs have also been noted between people living in the same country. For instance, the levels of MePB in human hair from Spain varied from 10.2 to 33 ng/g^38^, and according to another observation it ranged from 68.3 to 14,187 ng/g^14^. These facts, together with clear differences in particular persons included into previous studies^14,39^ and the present investigation strongly suggest that the degree of exposure to PBs depends on various factors in the immediate human environment. These factors are not fully specified, but may include among others the frequency of cosmetics use, food, medications, profession and/or home furnishing. The concentrations noted during the present study were relatively high, although Olsztyn, where this study was performed, is not a highly industrialized region. So, high levels of the PBs noted in this study are not related to the degree of industrialization and the resulting pollution of the environment.

Up to now, only a few studies concerning biomonitoring of human exposure to PBs through hair sample analysis have been published (Table 2).

**Table 2:** Ranges and means (in brackets) of the concentration of parabens in human hair samples (pg/mg) noted in previous observations and the present study.

Country	n	MePB	EtPB	PrPB	BePB	BuPB	References
Germany	4		810–1980 (1365)	400–1520 (940)			^40^
Greece	95	17.6–27,437.0 (4501.2)	11.0–4224.5 (510.1)		2.1–66.6 (22.9)	1.8–2513.7 (237.1)	^13^
Korea	10	48.3–224.2 (123.6)	11.5–158.3 (64.5)	70.2–214.5 (136.9)	15.3–100.2 (55.6)	25.4–111.1 (74.2)	^41^
Spain	6	78–624 (246.5)	7.0–42 (19.83)	27–238 (140.83)			^42^
	6	10.2–33 (20.7)	9 (9)	11.6–107 (66.9)		3.5–9.4 (5.76)	^38^
	42	68.3–14,187 (2820.7)	2.9–6565 (634.8)	12.5–9009 (1006.1)			^14^
Poland	30	87.2–42,430.1 (4302.0)	19.1–8413.1 (704.0)	18.9–11,150.3 (825.7)	36.0–700.7 (135.2)	3.8–2636.0 (154.5)	Current study

*n* number of samples included into the study.

The values noted in the present study in comparison to previous observations (Table 2) are higher. It is best proven by the fact that the maximum concentration for all PBs studied noted in the present observation have not yet been described at this level. This phenomenon is most visible in the case of BePB (Table 2). However, the present study confirms previous observations (Table 2) that hair analysis is a suitable method to evaluate human exposure to PBs and may be used instead of the analysis of “classic” matrices, such as urine or blood. Undoubtedly, ease of collection and storage of samples speak for the use of hair analysis.

Moreover, the present results confirm two aspects connected with human exposure to PBs. The first of them is the fact that PBs with short-chain functional groups (such as MePB, EtPB and PrPB) are present in the human organism in the highest concentrations, and therefore their adverse effects may have the most important influence on human health status. This is because such parabens are the most commonly used in the industry, which leads to relatively high environmental pollution with these substances^1,20^. The second observation is that the human body is exposed simultaneously to some PBs. This fact, noted both in the present study, where exposure to at least three PBs was noted in all samples and in previous observations^13,14,38^, is very important in terms of toxicology. It is relatively well known that exposure to several chemicals may have adverse effects different from exposure to a single substance. This is because of interactions between chemicals, which may lead to a possible synergistic or additive effect^4,20,43^. It should be remembered that such a situation may occur not only between particular PBs, but also between PBs and other endocrine disruptors commonly found in the environment, such as bisphenol A, triclosan or polyfluoroalkyl substances^14,44–46^. Therefore, even moderate levels of single PBs, but with the simultaneous presence of other PBs and other types of endocrine disruptors, may affect human health. This is all the more likely since multidirectional adverse effects of PBs have been reported by an increasing number of studies^11,17–21^, and many aspects connected with the toxic activity of these substances are still not fully understood.

Differences in the concentration of PBs between men and women and between younger and older persons noted in the present study were not statistically significant. In the light of previous studies, it is known that higher levels of BPs are present in women^14,26–28,47^. This phenomenon is explained by the fact that women use a high number of cosmetics containing parabens not used by men (make-up, creams and mouthwash). On the other hand, previous observations have shown that men are also to a large extent exposed to PBs by the use of cosmetics^48–50^, and no statistically significant differences in PB levels between men and women were observed in the present study, which confirms the previous observations. Moreover, previous studies have found that concentrations of PBs in older humans are higher than in younger people^14^, due to the fact that older people most often use personal care products. The present results support this thesis, but only partially. Although the mean level of most PBs studied were higher in older persons in comparison to young adults, the differences between these two groups were not statistically significant.

Moreover, during the present study no statistically significant differences in the concentrations of PBs were noted between persons with colored hair and persons with uncolored hair. It proves that the proper preparation of hair samples (mainly thorough washing in ultrapure water and methanol) removes any external contamination from the hair, and therefore hair sample analysis is a suitable method of biomonitoring human exposure to PBs, which is in agreement with previous observations^13,14^.

However, this study has some limitations. The first is the number of volunteers included into the experiment. Although many previous studies on exposure to organic pollutants have been conducted on similar or even smaller populations^31,41,51,52^, it is commonly known that the larger the study group, the more representative the results will be. For this reason, the present study may be considered preliminary, and the exact monitoring of human exposure to PBs in various regions of Poland requires further research. The next limitation is connected with the fact that fur analysis takes into account both external and internal exposure, and the separation of externally deposited compounds from endogenously deposited substances is difficult. External exposure may depend on how long the hair was on the head (distance from the scalp). Although the hair samples in this study were collected from the same place on the head near the scalp, the hairs had various length (depending on the hairstyle). This fact may have had an influence on the levels of PBs, because longer hair is exposed to environmental factors for longer, which may lead to higher concentrations of substances studied. On the other hand, during the present study there were no strict correlations between hair length and PB levels (Tables 1, 3). For example, in sample number P17 (hair length 0.5 cm) the concentration was extremely higher than in sample P10 (hair length 12–14 cm). Nevertheless, this study showed human exposure to PBs, and the exact description of the correlation between hair length and concentration of PBs needs further studies.

**Table 3:** Characterization of volunteers taking part in the study.

No	Age	Gender	Colour	Hair coloring	Length (cm)
1	46	Male	Black	No	1–2
2	45	Male	Black	No	3–4
3	28	Female	Brown	Yes	3
4	23	Male	Black	No	8–9
5	32	Male	Black	No	2–3
6	34	Male	Black	No	1
7	50	Female	Blond	Yes	3–4
8	53	Female	Black	Yes	3–4
9	28	Male	Black	No	3–4
10	22	Female	Blond	No	12–14
11	27	Female	Black	No	7–9
12	24	Female	Brown	No	7
13	23	Female	Brown	No	7
14	23	Male	Brown	No	0.5
15	61	Male	Black-Gray	No	1
16	35	Male	Black	No	1
17	55	Male	Black-Gray	No	0.5
18	49	Male	Black	No	6–7
19	23	Male	Brown	No	11
20	22	Female	Black-Brown	Yes	5
21	56	Female	Brown	Yes	3–4
22	55	Female	Brown–Red	Yes	5–6
23	48	Female	Brown	Yes	3–4
24	46	Female	Black	Yes	8–10
25	50	Male	Grey	No	0.5
26	31	Female	Brown	Yes	–
27	27	Female	Brown	No	6–7
28	67	Female	White	No	6
29	34	Male	Black	No	0.5
30	59	Male	Black	No	3

To sum up, the obtained results have shown that inhabitants of Olsztyn and the surrounding area (northern east Poland) are simultaneously exposed to various PBs. Simultaneous exposure to some PBs is important in terms of toxicology, because substances may interact with each other. Such a situation may lead to a possible synergistic or additive adverse effect on the human organism. The presence of PBs strongly suggests that these substances have an influence on human health. Moreover, the present study confirms previous observations that analysis of the hair samples is a suitable method for the biomonitoring of human exposure to PBs. However, the above mentioned limitations of this study suggests that the exact explanations of all the aspects connected with the presence of PBs in human hair in Poland requires further study.

## Materials and methods

### Materials

MePB, EtPB , PrBP , BuPB, BePB (all ≥ 99%), and ammonium acetate (≥ 98%) were purchased from Sigma-Aldrich (St. Louis, MO, USA), methanol (LC–MS grade) from Honeywell–Riedel de Haën (Seelze, Germany), acetonitrile (LC–MS grade) from Fisher Chemical and phenobarbital-d^5^ from Isotec Inc. (Miamisburg, OH, USA). Ultrapure water was produced by Merck's Direct-Q 3UVwater purification system (Darmstadt, Germany).

### Sample collection

Head hair samples were collected from 30 volunteers—adult persons of both genders (15 women and 15 men), aged between 22 and 67, residents of Olsztyn (city in north-eastern Poland) and the surrounding area. Information about volunteers are shown in Table 3. Before collection of samples all people were informed about the study and agreed to the sampling procedure. Sampling collection was performed according to the agreements of the Bioethical Committee at the School of Medicine of the University of Warmia and Mazury in Olsztyn (Poland) (agreement numbers: 27/2017 and 5/2021). The studies were performed with informed consent. All experimental methods were applied in accordance with the relevant guidelines and European and Polish regulations. Hair samples were collected from the back of the head above the nape of the neck, near the scalp, and differences in the hair length were due to the different hairstyles of the volunteers. Immediately after collection, hair samples (about 2 g) were wrapped in aluminum foil and stored in the dark at room temperature until further studies. During storage, samples did not come into contact with materials containing parabens.

### Extraction of PBs from hair

Hair samples were washed four times (twice with ultrapure water and twice in methanol) to remove external contamination from the hair. Then the hair samples were dried and cut into small pieces. The extraction was performed according to the method previously described by Tzatzarakis et al.^53^. In short, 100 mg of each sample were put into glass screw tubes, treated with 2 ml of methanol and subjected to extraction in an ultrasonic water bath for 4 h with periodic mixing with a vortex system. After centrifugation, the extract was put in glass tubes and evaporated to dryness under nitrogen steam at 35 °C. Then 100 μl of methanol was added to residues and the solution was transferred into 2 ml vials for liquid chromatography–mass spectrometry (LC–MS) analysis.

### Instrumentation

Analysis was performed with a Shimadzu (Kyoto, Japan) liquid chromatography–mass spectrometry system (LC–MS 2010 EV) equipped with an autosampler. Analyte separation was performed using a Supelco Discovery column C18 (250 mm, 4.6 mm, 5 μm) (Sigma-Aldrich, St. Louis, MO, USA) at 30 °C stable oven temperature. A gradient of 5 mM ammonium acetate (solvent A) and acetonitrile (solvent B) were chosen for the analysis with a flow rate 0.6 ml/min. Atmospheric pressure chemical ionization (APCI) combined with a quadrapole mass filter in a selected ion monitoring (SIM) negative mode were used for monitoring the aforementioned substances.

### Method validation

To ensure the efficacy of the method used in the present study, analytical parameters were checked. Standard solutions of the analytes were performed in concentrations of 0, 50, 100, 250 and 500 ng/ml. Linearity of the standard solutions was found to be 0.999 for MePB, 0.9985 for EtPB, 0.9969 for PrPB, 0.9997 for BePB and 0.9988 for BuPB. Spiked samples were also performed in concentrations of 0, 50, 100, 250 and 500 pg/mg. Linearity was found at 0.9987 form MePB, 0.9867 for EtPB, 0.9979 for PrBP, 0.9920 for BePB and 0.9945 for BuPB.

Limit of detection (LOD) and limit of quantification (LOQ) were calculated using the signal to noise ratio. Recovery, accuracy and inter day precision (%RSD) of the method were examined using 3 repeats of spiked samples (n = 3) for each above-mentioned tested concentration (50, 100, 250 and 500 pg/mg) (Table 4).

**Table 4:** Validation parameters of the applied methodology.

n = 3	Methyl paraben	Ethyl paraben	Propyl paraben	Benzyl paraben	Butyl paraben
Mean % recovery	125.0	90.7	99.7	83.4	107.0
± SD	4.4	16.3	8.2	15.9	12.4
Mean % accuracy	102.2	105.8	109.7	84.5	109.6
± SD	3.3	20.4	11.3	14.9	14.6
Precision (% RSD)	15.8	12.7	14.0	21.9	9.5
± SD	4.7	6.9	10.6	2.9	4.2
LOD (pg/mg)	0.4	1.0	0.7	1.1	0.2
LOQ (pg/mg)	1.4	3.3	2.2	3.6	0.8
r^2^ (spiked curves)	0.9987	0.9867	0.9979	0.9920	0.9945
r^2^ (standard curves)	0.9990	0.9985	0.9969	0.9997	0.9988

### Statistical analysis

The statistical analysis was performed using GraphPad Prism version 9.2.0 (GraphPad Software, San Diego, California USA). In the case of a comparison between two groups (males versus females, younger volunteers versus their elders, persons with colored hair versus persons with natural hair color) non parametric Mann–Whitney test was used. The differences were considered statistically significant at *p* < 0.05.
